# Mapping the Mechanome of Live Stem Cells Using a Novel Method to Measure Local Strain Fields *In Situ* at the Fluid-Cell Interface

**DOI:** 10.1371/journal.pone.0043601

**Published:** 2012-09-10

**Authors:** Min Jae Song, Susann M. Brady-Kalnay, Sara H. McBride, Polly Phillips-Mason, David Dean, Melissa L. Knothe Tate

**Affiliations:** 1 Department of Biomedical Engineering, Case Western Reserve University, Cleveland, Ohio, United States of America; 2 Department of Molecular Biology and Microbiology, School of Medicine, Case Western Reserve University, Cleveland, Ohio, United States of America; 3 Department of Neurological Surgery, Case Western Reserve University, Cleveland, Ohio, United States of America; 4 Department of Mechanical & Aerospace Engineering, Case Western Reserve University, Cleveland, Ohio, United States of America,; University of Zurich, Switzerland

## Abstract

During mesenchymal condensation, the initial step of skeletogenesis, transduction of minute mechanical forces to the nucleus is associated with up or down-regulation of genes, ultimately resulting in formation of the skeletal template and appropriate cell lineage commitment. The summation of these biophysical cues affects the cell's shape and fate. Here, we predict and measure surface strain, in live stem cells, in response to controlled delivery of stresses, providing a platform to direct short-term structure - function relationships and long-term fate decisions. We measure local strains on stem cell surfaces using fluorescent microbeads coated with Concanavalin A. During delivery of controlled mechanical stresses, 4-Dimensional (x,y,z,t) displacements of the bound beads are measured as surface strains using confocal microscopy and image reconstruction. Similarly, micro-particle image velocimetry (μ-piv) is used to track flow fields with fluorescent microspheres. The measured flow velocity gradient is used to calculate stress imparted by fluid drag at the surface of the cell. We compare strain measured on cell surfaces with those predicted computationally using parametric estimates of the cell's elastic and shear modulus. Finally, cross-correlating stress - strain data to measures of gene transcription marking lineage commitment enables us to create stress - strain - fate maps, for live stem cells *in situ*. The studies show significant correlations between live stem cell stress - strain relationships and lineage commitment. The method presented here provides a novel means to probe the live stem cell's mechanome, enabling mechanistic studies of the role of mechanics in lineage commitment as it unfolds.

## Introduction

After just 11.5 days in the womb, before the first twitch of skeletal muscle, cells of the developing mouse limb bud experience a life-changing event. Termed mesenchymal condensation, this transformative event is the first step in skeletogenesis. Though the exact timing of mesenchymal condensation varies between vertebrate species, prior to condensation, every cell in the developing mesoderm shares not only common DNA but also a common, undifferentiated phenotype, rendering multipotency. Furthermore, prior to this time in development, diffusive transport is efficient to insure cell viability in the tiny limb template. However, from this time point onwards, the cells of the limb bud begin to self assemble and to specialize their function while forming the specific tissues of the musculoskeletal system, including *e.g.* bone, cartilage, fat, vascular tissue and muscle [1], [2]. The subsequent exposure of stem cells to spatially and temporally varying biophysical and chemical signals guides the cells to specialize their structure for prevailing function, or to commit to a specific lineage. In this way, “form emerges from function in the stem cell's mechan[o-chemo-biolog]ical world” [1], [3].

**Figure 1:**
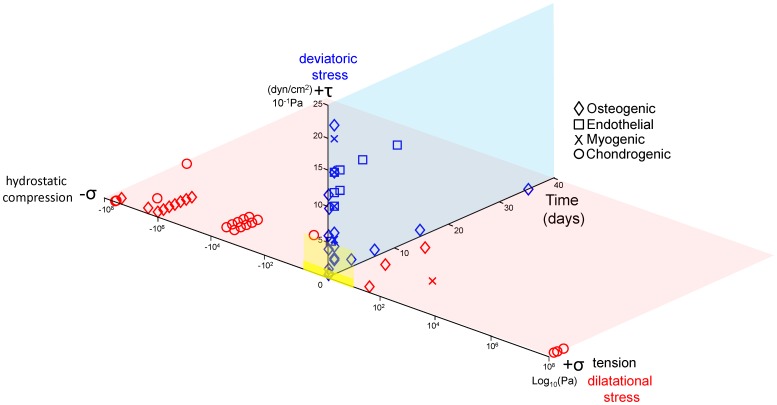
Characteristic magnitudes and time domains of mechanical signals applied in studies of multipotent cell differentiation. Each data point represents one study. The shape of the data point portrays the lineage to which the multipotent cell committed. Blue data points depict deviatoric (shape changing) stresses, i.e. shear stress magnitudes [dyn/cm^2^], and duration of signal over the time course of the study [days]. Red data points depict dilatational stresses (volume changing stresses, *i.e.* hydrostatic compression and tension, [log_10_Pa]). The yellow plane (opaque to transparent reflecting respective likelihood of the stress ranges) overlay represents dilatational and deviatoric stress ranges predicted during cell fate determination in utero [5], [13], [15]–[43].

The chemical cues to generate targeted gene transcription typical for lineage commitment to specific cell fates are well understood. In fact, differentiation media to achieve targeted fates are commercially available [1], [4]. However, no such protocol or reference library exists to guide stem cell differentiation using mechanical cues [1], [5]. Furthermore, although many published studies have addressed structure – function relationships in terminally differentiated cells [6]–[14] or in stem cells at mid to late stages of embryonic development, where vascular pressure gradients and/or muscle forces can either be measured or estimated, only recently have scientists begun to elucidate the role of mechanical forces at either the earliest stages of fate initiation or in live stem cells (Fig. 1, Table 1) [5], [13], [15]–[43]. Several recent studies show how exquisitely sensitive pluripotent cells are to the endogenous, mechanical signals of their own environment as well as to controlled, exogenously applied signals [3], [4], [31], [44]. Interestingly, stem cells do not possess the specialized surface proteins and structures exhibited by terminally differentiated cells to sense and transduce extracellular mechanical stimuli, necessitating other mechanisms for mechanotransduction. [4] Hence, the spatial and temporal unfolding, mechanotransduction mechanisms, as well as the plasticity of cell fate determination have yet to be elucidated, in part due to the challenge of controlling the applied stresses while measuring cell scale strains *in situ* and in live cells. This challenge provided the impetus for our current study, where we **developed methods to probe the stem cell's “mechanome,” enabling for the first time to our knowledge the elucidation of structure - function relationships and unfolding lineage commitment in live model embryonic mesenchymal stem cells**.

**Table 1:** Magnitude and time of mechanical stresses applied in stem cell differentiation studies.

Lineage	Duration [day]	Magnitude [dyn/cm^2^]	References
Osteogenic	4	1	Datta *et al.* 2006
	8	1	
	16	1	
	1	6	Knippenberg *et al.* 2005
	1	2	
	0.1	0.3	Billotte *et al.* 2004
	0.01	4	Bakker *et al. 2000*
	0.01	6	Bakker *et al.* 2001
	0.01	12	Bakker *et al.* 2002
	1	4	Yourek *et al.* 2010
	0.04	0.2	McBride *et al.* 2008
	0.04	1	
	0.1	10	Arnsdorf *et al.* 2010
	1	2.3	Kreke *et al.* 2008
Endothelial	6	15	Wang *et al.* 2005
	0.3	5	Nikmanesh *et al.* 2012
	2	15	Ahsan *et al.* 2010
	1	10	Bai *et al.* 2009
	1	15	
	2	12	Zhang *et al.*2011
	1	12	Wu *et al.*, 2008
	0.5	5	Ye *et al.* 2007
Myogenic	1	5	Huang *et al.* 2010
	1	10	
	1	15	
	1	20	

Our goal was to develop a novel method to measure strain at the interface between the cell and its environment concomitant to delivery of controlled mechanical cues and assessment of cell fate. For this purpose, similar to the use of reflective optical tracking of markers in human gait studies or speckles in tissue mechanics studies [45], we tracked point displacements of microbeads coated with a protein that targets the glycoproteins on the cell surface. We first adsorbed Concanavalin A (Con A), a lectin and carbohydrate-binding protein, to the surface of 1 µm diameter microbeads. The Con A-protein coated microbeads then bound to the naturally occurring glycoproteins of the stem cell's surface coat (glycocalyx). To apply stress fields to cells in a controlled manner, we seeded the cells at different target densities (shown previously to effect dilatational or volume changing stresses [3]) and then exposed them to controlled fluid drag forces (shown previously to induce deviatoric, shape changing, and dilatational stresses [4], [46]) in a flow chamber designed for this purpose [47], [48]. We used our previously established μ-PIV methods to visualize flow and to track, in four dimensions (4D, x,y,z,t) and at a sub-cell length scale, the stresses applied at cell boundaries by flow-induced fluid drag [46]. Then we tracked cell strain in the xy and z planes using confocal microscopy. Hence, we used combination of novel computational and experimental methods to predict and to expose the live stem cells to shape (deviatoric) and volume (dilatational) changing stresses while measuring cell scale strains *in situ*, in live cells. We then cross-correlated these variables (stress, strain) to measurements of gene transcription typical for pre-, peri- and post-mesenchymal condensation in cells exposed to identical protocols. In sum, we predicted and probed the stem cell's mechanome in living cells at very early stages in development.

## Materials and Methods

### Cells

A model mesenchymal stem cell (MSC) line, derived from the mesenchyme of murine embryos (C3H/10T1/2 cell line, CCL-226; ATCC, Manassas, VA), was used; these cells do not show the phenotypic drift that we observed previously in primary murine cells derived from the mesoderm at the time of condensation [1]. Furthermore, these cells exhibit multipotency, *i.e.* the capacity to differentiate along osteogenic, chondrogenic, adipogenic, smooth muscle [49], [50], and endothelial lineages [44]. Cells were passaged until P5 or P6, according to published protocols, and seeded at densities including 0 (control), 16,500 (low density), 35,000 (high density), and 86,500 (very high density) cells/cm^2^, measured using a hemocytometer [44].

Three glass coverslips were prepared for each density. Previous [47], [51] and pilot studies showed that cells seeded near fluid entry and exit portals disrupt flow. Hence, in order to mitigate these effects, each glass coverslip was first covered with a silicon sheet (BioPlexus, Ventura, CA) punched in the center with a 3 mm diameter hole, which provided an uncovered area of the coverslip onto which cells were then seeded. Three hours thereafter, the silicon sheet was removed. Cells were incubated for 24 hours total prior to dilatational and deviatoric stress exposure. Cells were stained with calcein, which allowed for real-time imaging of cells concomitant to strain and flow field mapping as well as demonstrating cell viability throughout testing.

### Probing the live stem cell's mechanome

Cells were subjected to controlled and measureable stress fields using fluid flow [46] and seeding protocols [3]. Cells were subjected to controlled and measureable stress fields using fluid flow. Strain on cell surfaces was measured *in situ* by tracking the displacement of microbeads bound to glycoproteins on the cell surface. Precise flow regimes were traced by tracking the displacement of microspheres placed in the flow field over time (using μ-piv methods). In this way, stress - strain curves could be created for cohorts of cells. These mechanical property datasets were then related to cell lineage commitment by cross correlating applied stresses and resulting strains to up- and down-regulation of gene transcription data from a separate study [31], [44] in which cohorts of cells were treated identically to the cells of the current study.

### Preparation of and tracking of microbeads to map strain on cell surfaces

One µm diameter microbeads were coated with a protein that targets the glycoproteins on surface of the cell, allowing for binding of the microbeads to the cell surface. First, Concanavalin A (Con A), a lectin and carbohydrate-binding protein, was adsorbed to the microbead surface (Polysciences, Inc., Warrington, PA) using a protocol adapted from the manufacturer. A solution containing the Con A-protein coated microbeads (2.275×10^10^ microbeads/ml) was introduced to the cell culture medium (50 µl microbead solution per well); the Con A on the bead surface bound to the naturally occurring glycoproteins on the surface of the seeded stem cells.

**Figure 2:**
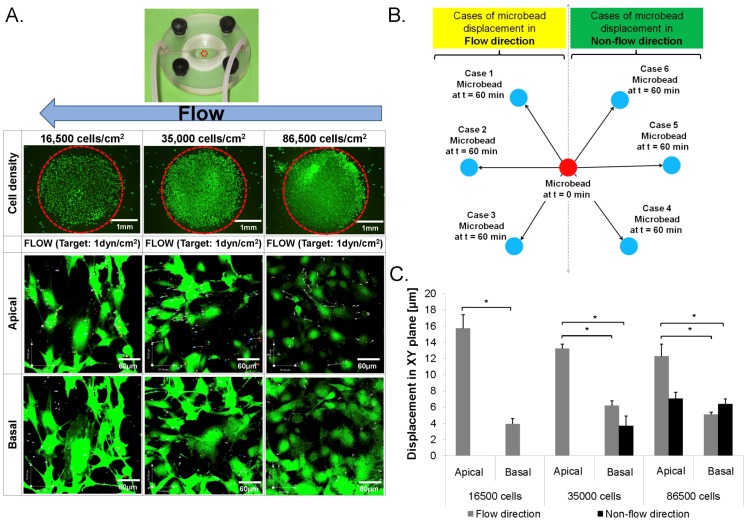
*In situ* mapping of stem cell stresses and strains. 4D (x,y,z,t) image of microsphere displacements (**A, C, E**) and microbead displacements (**B, D, F**) shows flow fields and surface strains, respectively, in live stem cells subjected to fluid flow at low (**A,B**), high (**C,D**), and very high (**E,F**) densities. Calcein Green stains live cells. Red and white arrows indicate velocity of flow (microsphere displacement and direction: **A, C, E**) and strain (microbead displacements: **B, D, F**), respectively. Red, green, and white dots (**B, D, F**) show respective microbead positions at 0, 30, and 60 minutes after flow. Stresses imparted by flow at cell surfaces are calculated from the experimentally determined velocity gradient (slope of Fig. S1, Equation 3). Cell surface strains (deformations) are calculated using experimentally measured microbead displacements on cell surfaces.

During application of controlled flow fields, we imaged displacements of these microbeads in the XY plane to determine their displacements during exposure of the cell surface to flow-induced drag forces. Confocal images were taken every 10 minutes during exposure to the target shear stress (1 dyn/cm^2^, 0.657 ml/min, Fig. **2** **,** **3**). Displacements in the Z direction (height) were tracked as a function of fluorescence intensity and microbead spatial pattern.

**Figure 3:**
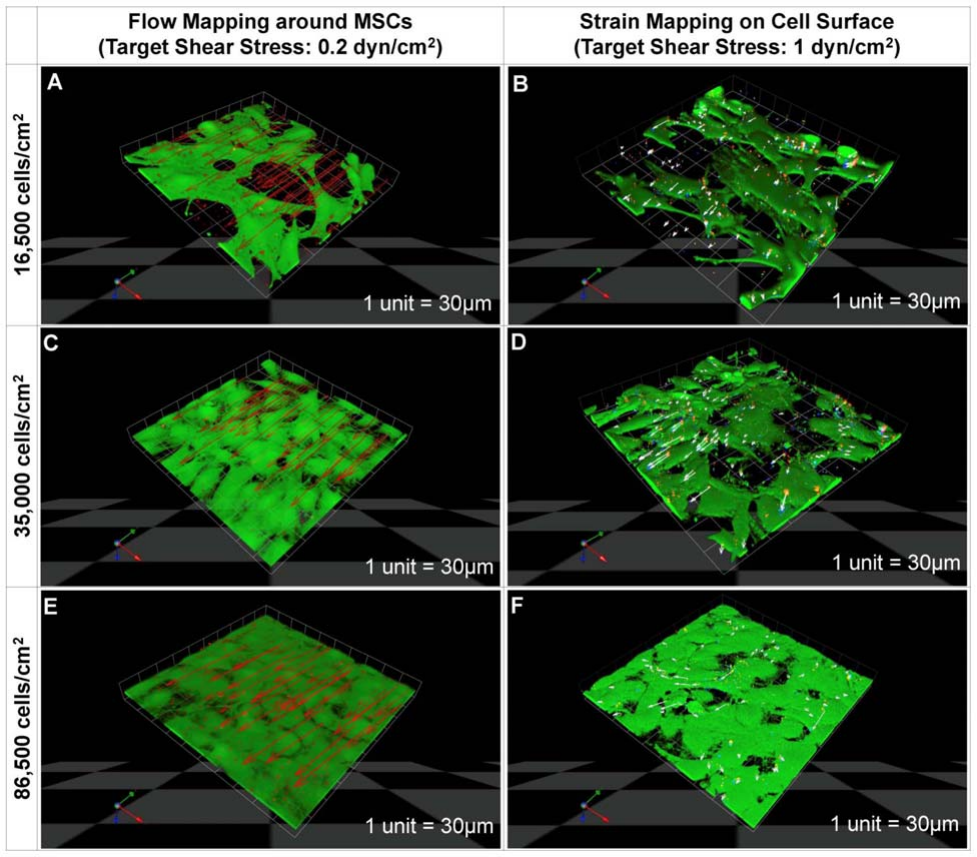
Computational predictions (CFD-FEM) of strains on cell surfaces for flow rates imparting 0.2 (A,B) and 1 dyn/cm^2^ (C,D). Each computational prediction includes velocity magnitude and displacement in XY plane, assuming an elastic modulus of 0.9 Pa (**A** and **C**), and in the Z direction, assuming an elastic modulus of 90 Pa (B and D) at 16,500 cells/cm^2^ (**1**); computational predictions were carried out for 35,000 cells/cm^2^ (**2**), and 86,500 cells/cm^2^ (**3**) cell seeding densities. Topological color map indicates displacements on surface of seeded cells. The geometry of the seeded cells is imported from a 3D reconstruction of confocal images. Color arrows show flow velocity (flow field) in the near the vicinity of cells.

### Micro-particle image velocimetry (μ-piv) to visualize and measure flow fields around cells

**Figure 4:**
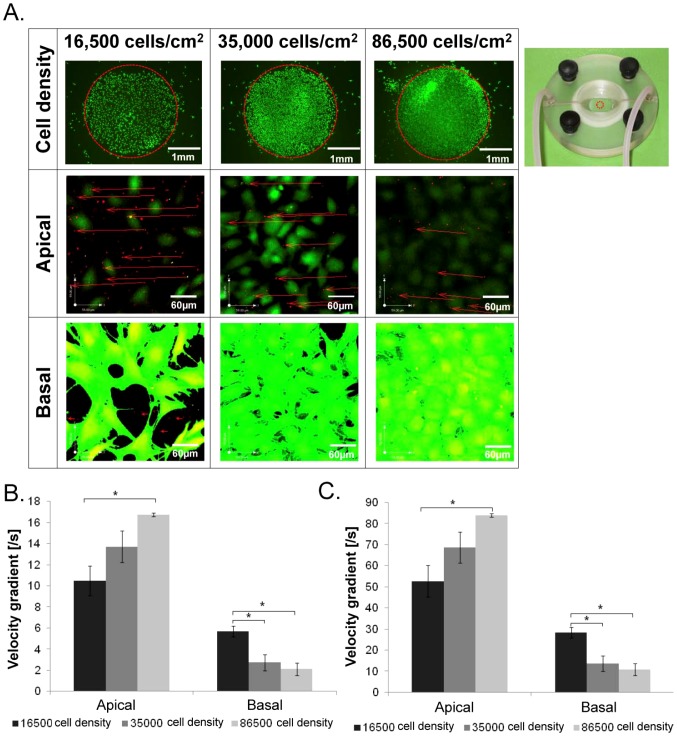
Mechanical testing (stress - strain analysis) of live stem cells using novel microbead tracking method. Cell surface deformation (strain) is mapped *in situ* concomitant to delivery of controlled mechanical cues via fluid flow. (**A)** **Two dimensional (2D) images of microsphere displacements on cell surfaces.** Calcein green stain marks live stem cells. Red, green, and blue dots indicate respective positions of microbeads at 0, 30, and 60 minutes after flow application. Control shows positions of microbeads in absence of flow. (**B**) Schematic depiction of flow and non-flow orientation/direction with respect to initial position of microbead (t_0_, red). (**C**) Comparison of microbead displacements on cell surfaces from control cohorts and cohorts exposed to flow. Error bars show standard errors (n = 3), * indicates statistical significance (p<0.05).

Mechanical cues were delivered to cell surfaces using a flow chamber, which was custom designed to deliver highly controlled mechanical cues (flow fields) to cells seeded within, while allowing for *in situ* confocal imaging. [48] The 1 cm (width)×2.3 cm (length)×250 µm (height) flow chamber gasket defines the flow field dimensions (Fig. **4**). The flow chamber was fixed in one position while μ-PIV tests (Fig. **2** **,** **5**) were carried out, using our published protocols, to track and measure drag forces at interfaces between cell surfaces and the flow field. [46] Confocal images were taken within a 4 mm×4 mm area defined by fields of view chosen as representative for each density group (40× objective, SP2 laser scanning confocal microscope, Leica Microsystems, Mannheim, Germany). Images were acquired at 256×256 resolution over 990 ms. Microsphere (Invitrogen Life Technologies, Carlsbad, CA) displacements were tracked (Image J, NIH, Bethesda, MD) and velocity vectors calculated (Volocity, Perkin Elmer, Inc., Waltham, MA), after which shear stresses were calculated based on Newtonian fluid mechanics theory. Flow was controlled using a syringe pump (Harvard Apparatus, Holliston, MA) and the flow rate, 0.132 ml/min, which was calculated using computational fluid dynamics (CFD) for the delivery of the target shear stress (0.2 dyn/cm^2^, measured on the substrate surface in the absence of cells).

**Figure 5:**
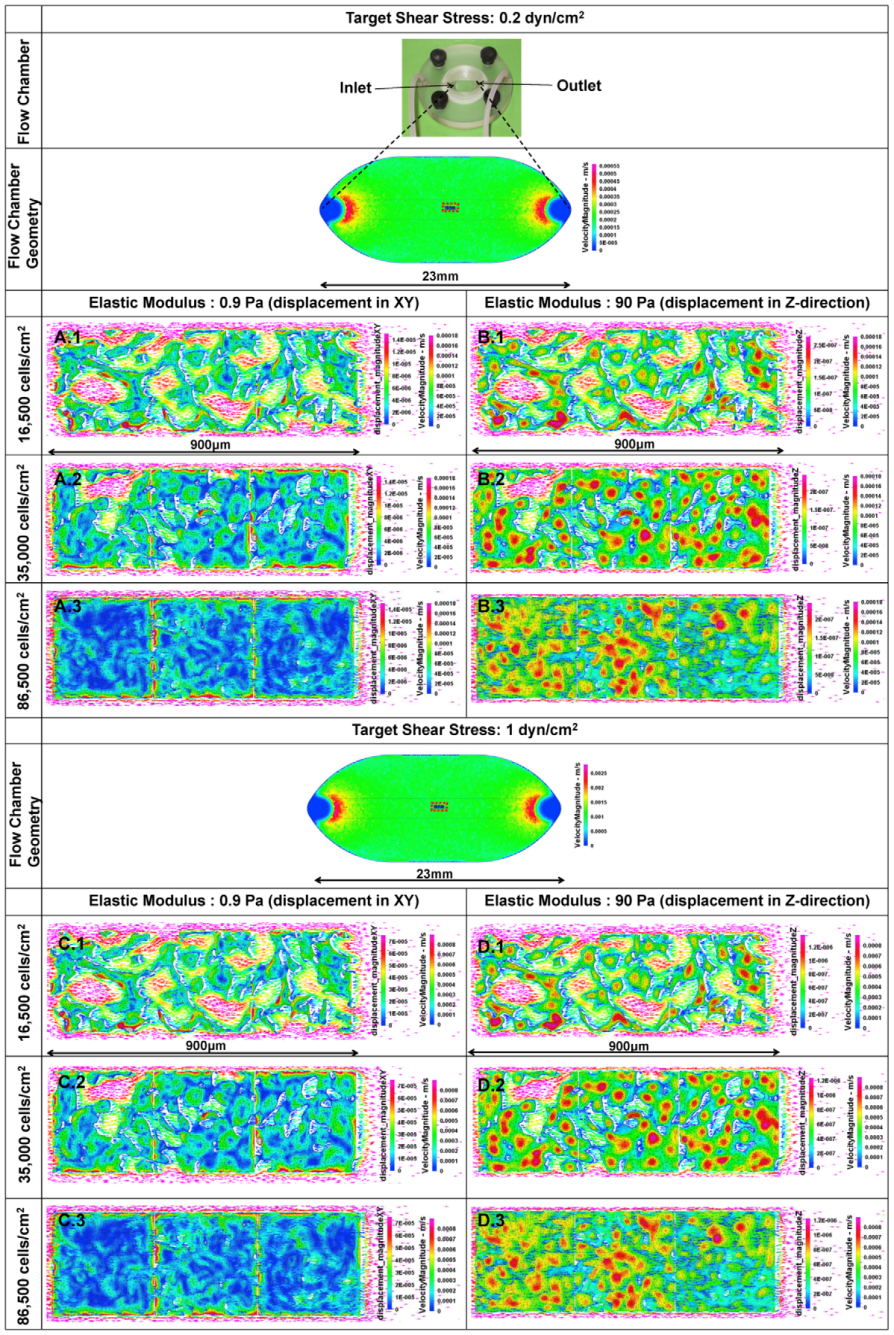
Experimental and computational analysis of flow regimes around live stem cells. (**A**) 2D micrographs of μ-PIV experiments show flow regimes in the apical and basal regions of the live stem cell (target shear stress imparted by flow: 0.2 dyn/cm^2^). (**B,C**) Validated computational fluid dynamics (CFD) predictions of velocity gradients (which drive shear gradients and hence target shear stresses) in the apical and basal regions of cells, at flow rates including 0.2 and 1 dyn/cm^2^, respectively. * indicates statistical significance, defined by p<0.05. Error bars show standard errors (n = 3).

### Parameter Estimation using Computational Models

A partially coupled computational fluid dynamics (CFD) – finite element method (FEM), multiphysics (solid-fluid) model (CFD-ACE, ESI Group) (Fig. **4**) was developed to elucidate stress and strains on cell surfaces (cell-cell and cell-fluid interfaces) induced by flow. Flow was assumed to be laminar and the fluid was idealized as Newtonian with 996 kg/m^3^ density, 310 K (body temperature), and 0.001 kg/ms viscosity. Cells were idealized as exhibiting elastic material properties.

Theoretically, the elastic modulus of the stem cell may vary between specific stem cell types (e.g. embryonic versus cells derived from mature subjects, pluripotent versus multipotent, etc.). Hence we used parametric estimation to determine elastic modulus of the live stem cells of this study. Using our computational model, we first predicted cell behavior for elastic moduli of different orders of magnitude, varying the elastic modulus from 0.9, 9, to 90 Pa. The estimated range of moduli was chosen based on our recent studies exhibiting up to a 1000 fold mechanosensitivity of stem cells compared to terminally differentiated cells [5], as well as published reports on the elastic modulus of osteoblasts (900 Pa) [52]. The Poisson's ratio was assumed to be 0.36, based on previously reported measures in chondrocytes [53]. By comparing predicted stress-strain data with actual (measured) data, we were able to estimate the actual modulus of elasticity for the model stem cell line.

Input pressure gradients were applied to the model flow chamber, comprising 4.0 Pa to achieve a target 0.2 dyn/cm^2^ and 20 Pa to achieve a target 1 dyn/cm^2^ shear stress (τ), shown in previous studies to elicit significant up- and down-regulation of baseline gene activity and used previously in our *in situ* micro-PIV studies in live stem cells [4], [31]. The Navier-Stokes equation was applied, assuming that body forces were negligible and that flow was steady in three dimensions, also appropriate assumptions for the length and time scale as well as the flow velocity studied [5], [46]. Hence,
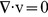
(1)

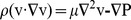
(2)

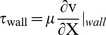
(3)where 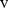 is the velocity vector, 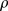 is density, 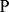 is pressure, 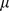 is viscosity, 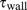 is the shear stress at the bottom of flow chamber, 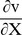 is the strain rate, and 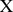 is the distance from bottom of chamber. Linear elastic theory was applied with the finite element method to elucidate cell scale stresses and strains,
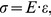
(4)where E is an elastic modulus, and σ, ε indicate stress and strain, respectively. Thereafter, CFD and FEM were partially coupled to elucidate how boundary conditions change with shear stress magnitude under steady state conditions.

CFD-FEM was modeled based on four-dimensional (4D: x,y,z,t) confocal microscope (Leica Microsystems GmbH, Mannheim, Germany) images of cells seeded at each density. Image stacks were acquired for three fields of view (each 300 µm×300 µm), chosen randomly for each of three trials carried out at each density. The three image stacks were reconstructed in 4D. The 4D reconstructions were then meshed using Amira (Visage Imaging Inc. CA) for transfer and analysis using computational fluid dynamics (CFD) software (CFD-GEOM, CFD-ACE, VIEW). For idealization purposes, three 300 µm×300 µm areas, representing together a rectangular area comprising 900 µm×300 µm at the center of flow chamber geometry, were investigated (Fig. **4**).

### Statistical Analysis

#### Parametric analysis of stem cell mechanical properties

**Figure 6:**
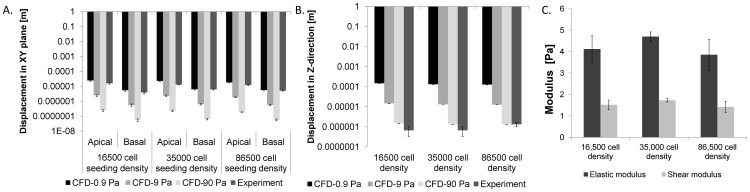
Parametric estimation of the stem cell's mechanical properties. Displacements in the XY plane (**A**) and Z direction (**B**) were predicted computationally, using values varied parametrically (by orders of magnitude) about a known measured value for osteoblasts (900 Pa). By comparing experimentally measured displacements with displacements predicted for model cells with estimated moduli, a best estimate for the elastic and shear moduli of the model stem cell line could be made. The vertical axes are depicted in a logarithmic scale. **C.** Through linear interpolation, parametric estimates were made for elastic and shear modulus of the model stem cell, for flow generating a target shear of 1 dyn/cm^2^ and for three seeding conditions (different densities). Error bars show standard errors (n = 3).

Parametric estimation of the elastic modulus for cells in each cohort (density group) was carried out using linear interpolation (Fig. 6C) of experimental data and computational predictions, *i.e.*

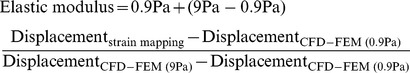
(5)The shear modulus was calculated using linear elastic theory (Equation 6),
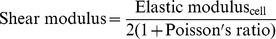
(6)The Poisson's ratio was estimated based on that of the chondrocyte (0.36), which is within the range of compressible media.

**Figure 7:**
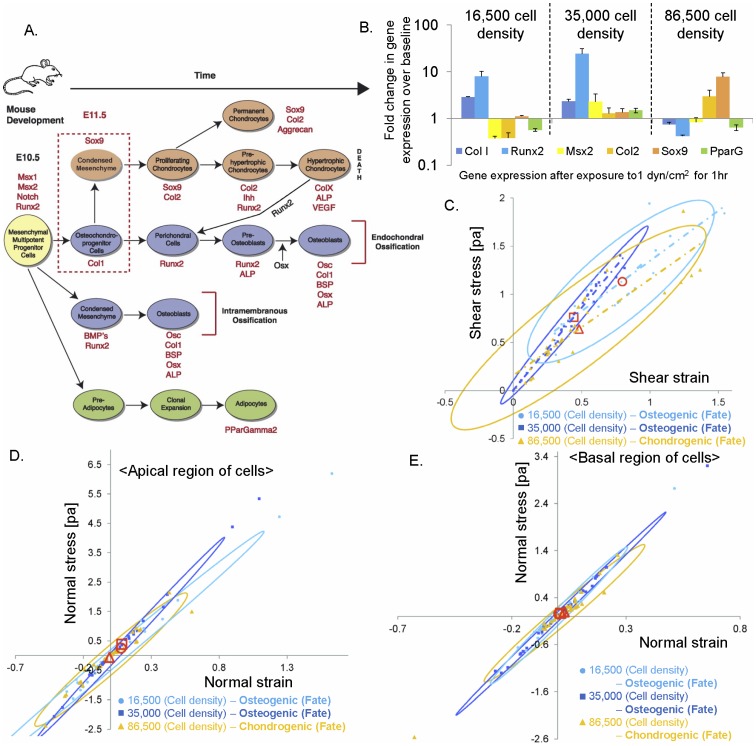
Stress - strain - fate plots to probe the live stem cell's mechanome, in a case study in live stem cells exposed to 1 dyn/cm^2^ shear stress via fluid flow. (**A**) After mesenchymal condensation (red dotted box, E11.5 in the mouse), the first step in skeletogenesis, stem cells follow lineage commitment paths toward chondrogenic (orange), osteogenic (blue), and adipogenic (green) fates. Transcription levels for specific proteins (red text) give a “chronological fingerprint” for each stage of development over time [1]. (**B**) For live stem cells exposed to 1 dyn/cm^2^ shear stress over 60 minutes, rtPCR data provide fold changes in gene expression of markers above or below baseline (no flow exposure) gene expression levels, based on analysis of previously reported data [31]. (**C**) Stress - strain - fate plot of live stem cells exposed to 1 dyn/cm^2^ shear stress over 60 minutes, where fate is indicated by color (osteogenic: blue, chondrogenic: orange). Linear regression of experimental data shows distinct slopes for each density and resultant fates, including osteogenic (blue: 1.04 [Pa], R^2^∼0.7, 16,500 cell density [cells/cm^2^]; navy: 0.9997 [Pa], R^2^∼0.78, 35,000 cell density [cells/cm^2^]), and chondrogenic lineage commitment (orange, 1.7124 [Pa], R^2^>0.8, 86,500 cell density [cells/cm^2^]). Red overlay shapes indicate mean strain stress at each density. Ellipses show 95% density area for each density. **D.** Local (apical), normal stress-strain (XY plane) relationship, depicting lineage commitment, including osteogenic (blue: 3.8513 [Pa] at 16,500 cell density [cells/cm^2^]; navy: 4.8513 [Pa] at 35,000 cell density [cells/cm^2^]) and chondrogenic lineages (orange: 3.8525 [Pa] at 86,500 cell density [cells/cm^2^]); all R^2^>0.8. Red shapes indicate mean stress and strain at each density. Ellipses indicate 95% density area for each density. **E.** Local (basal), normal stress-strain (XY plane) relationship, depicting lineage commitment, including osteogenic (blue: 4.9413 [Pa] at 16,500 cell density [cells/cm^2^]; navy: 4.8059 [Pa] at 35,000 cell density [cells/cm^2^]) and chondrogenic lineages (orange: 4.0653 [Pa] at 86,500 cell density [cells/cm^2^]); all R^2^>0.8. Each data point is shown from cohorts of cells in the field of view.

ANOVA (Tukey Kramer) tests were applied to determine significant differences between each modulus at each cell density (p<0.05, Minitab, State College, PA). Shear stress and normal stress for each group were calculated and plotted (Fig. 7C,D,E). 95% confidence and density ellipses were computed from the bivariate normal distribution fit to shear strain and stress variables. A linear regression was applied to obtain slopes from all groups at each cell density.

#### Validation of computational predictions using experimental data

For CFD-FEM predictions, 3D velocity and displacement data were averaged as a function of distance from the substrate, and standard errors were calculated (n = 3). For Accuracy (Fig. S1) of μ-PIV and strain mapping data, mean particle displacements and velocities were first measured for each image of a given image stack. Then, mean flow velocities were calculated for each layer, and from five samples at each cell density. Mean flow velocities and displacements were plotted with standard errors (n = 3). A linear regression technique was applied to the computational and μ-PIV data. Predictive power was calculated from the R^2^ value of the mean shear rate (linear regression of computational and μ-PIV data, R^2^>0.70 defined as significant). ANOVA (Tukey Kramer) tests were used to compare velocities and shear stresses for each pair of densities and locations (p<0.05 defined as significant differences, Minitab, State College, PA). Flow regimes around cells predicted by the multiphysics computational method showed more than 77% of accuracy compared to experimentally measured μ-PIV data (Fig. S1).

## Results

### Mapping strains on the surface of live stem cells

A strain mapping method was implemented to measure strains on the surface of live stem cells during exposure to 1 dyn/cm^2^ target shear stress (induced by flow at an optimized input flow rate, 0.657 ml/min, calculated using computational modeling methods described below). Microbead displacements were tracked (Fig. 4A) and measured (Fig. 4B,C) prior to (control) and during exposure to fluid flow. Spatiotemporal (4D: x,y,z,t) image reconstructions of microbead displacements allowed us to map local strain distributions on cell surfaces (Fig. 2B,D,F).

Strains on cell surfaces were highly dependent on not only exposure to flow *per se* but also to location, showing statistically significant differences with respect to distance from the substrate (basal: near substrate, apical: far from substrate) as well as the flow direction, respectively. There were significant differences between density cohorts, both in the apical and the basal region of cells. In all density cohorts, the greatest fold changes in bead displacement were observed in the basal region of cells, showing significant differences attributable both to density as well as flow direction.

### Tracking flow regimes in the near vicinity of live stem cells

The microparticle image velocimetry (μ-PIV) experiments showed displacements of microspheres in space and time, relative to the location of cells seeded within the flow chamber (Fig. **2**A,C,E, Fig. **5**A). By reconstructing in four dimensions (4D: x,y,z,t) the confocal image stacks obtained during μ-PIV testing, we could quantify precisely the flow regimes in the near vicinity of the live stem cells (Fig. **2**A,C,E, Fig. **5**B,C). These spatiotemporal reconstructions enabled calculation of velocity gradients which are used, with the Navier-Stokes equation, to calculate shear stresses exerted by fluid drag on cell surfaces.

### Computational modeling and parametric estimation of live stem cell mechanical properties

CFD-FEM predictions of flow fields for the lower target shear stress (0.2 dyn/cm^2^) were validated experimentally using μ-PIV methods, and CFD-FEM predictions of flow fields for the higher target shear stress (1 dyn/cm^2^) were compared with data from the parametric strain mapping study implementing three orders of magnitude of elastic modulus (0.9 Pa, 9 Pa, and 90 Pa). Comparing strain mapping data to CFD-FEM predictions, parametric estimation resulted in a stem cell elastic modulus between 0.9 and 9 Pa (Fig. 6A); linear interpolation gives an estimated elastic modulus close to 4 Pa (Fig. 6C). Based on displacements in the Z direction, which are affected mainly by hydrostatic pressure and cell seeding density, the MSC elastic modulus is closer to 90 Pa (Fig. 6B). In general, an approximately 1 µm displacement was observed in the Z-direction (Fig. 6B), although no statistically significant differences were observed between the different cell densities. Furthermore, there was no statistically significant difference in elastic modulus observed between densities.

Computational Fluid Dynamics-Finite Element Methods (CFD-FEM) were used to predict flow regimes and resulting displacements in a flow chamber, accounting for the absence or presence of cells exposed to either the 0.2 dyn/cm^2^ or 1 dyn/cm^2^ target shear stress. At 0.2 dyn/cm^2^, flow velocity and displacements in the XY plane and the Z direction were approximately five times smaller than those predicted for flows at 1 dyn/cm^2^. At approximately the mid-height of the cell, *i.e.* 17 µm from the bottom of the flow chamber, flow velocities were mapped based on CFD predictions (Fig. **3**). Similarly, displacements in the X, Y and Z directions were mapped (Fig. **3**,6), assuming the elastic moduli to be 0.9 Pa and 90 Pa based on the parametric study which showed that stem cells exhibit anisotropy (0.9 Pa for the input variables in the CFD-FEM model predicting fluid drag induced shear in the XY plane and 90 Pa for displacements in the Z direction). CFD-FEM predictions showed that, with increasing cell seeding density, flow velocity decreased. Furthermore, at very high cell density (86,500 cells/cm^2^), the flow space narrowed (Fig. **3**).

### Probing the stem cell's mechanome

Finally, we cross correlated stress - strain behavior of live stem cells, measured in this study, to measurements of gene transcription for early post-condensation markers of chondrogenesis, osteogenesis, and adipogenesis (Fig. 7A) [1] in cells exposed to identical protocols in a previous study [31], [44]. In that study, the fold change in gene expression of Runx2, an osteogenic marker, is significantly higher at high cell density (35,000 cells/cm^2^) and also high at low cell density (16,500 cells/cm^2^). The fold change in gene expression of Sox9, a chondrogenic marker, is significantly higher at very high cell density (86,500 cells/cm^2^) (Fig. 7A,B). Plotting of mechanical and lineage commitment data show that, at the target shear flow, both low and high densities exhibit markers typical of osteogenic lineage commitment whereas very high density cohorts exhibit markers typical of chondrogenic lineage commitment after exposure to one hour of 1 dyn/cm^2^ shear stress inducing fluid flow. Using the interpolated moduli and plotting local shear and normal stresses of seeded stem cells within the flow chamber (Fig. 7C), we observed significant correlation between stress - strain characteristics of cells and cell lineage commitment. With respect to stress - strain relationships, the stiffest slope is observed in cells seeded at high density and exposed to fluid flow. In contrast, the highest mean values of stress-strain are observed in cells seeded at low density and exposed to flow (Fig. 7C). Comparing to fold changes in gene expression of Runx2, the slope shows a higher correlation to the expression of Runx2 than does the magnitude of shear stress-strain to which the cell is exposed. Based on the local normal stress-strain relationship in the XY plane in apical areas (furthest from the substrate) cells seeded at low and high density exhibit spreading shape and tensile stress while cells seeded at very high cell density exhibit shrinking shape and compressive stress (Fig. 7D).

## Discussion

Probing the stem cell's mechanome is akin to carrying out mechanical testing on a material that exhibits not only viscoelastic properties and anisotropy but also whose phenotype is literally shaped by the test protocol. Stem cells comprise living, smart materials that adapt their structure and function in space and time to their mechanical *milieu*. Hence, even when subjected to stresses that would lie within the elastic range of the materials from which cells are made, the living cell exhibits a plastic response through adaptation (which itself is a response to both biochemically active processes of mechanotransduction as well as biochemically passive processes such as strain stiffening or stochastic processes of polymerization and depolymerization) and, ultimately, lineage commitment. A distinct advantage inherent to our method, which uses Con A coated microbeads to track strains on cell surfaces, is the ubiquity as well as the surface location (outer boundary of the cell) of the glycoprotein to which the Con A binds. A further advantage is the decoupling of microbead binding from active processes of mechanotransduction. For example, coating with a classical cadherin, an important transmembrane protein that is anchored by the actin cytoskeleton, would be tightly coupled to mechanotransduction processes.

In contrast to previous approaches that track emergent anisotropy of the actin and tubulin cytoskeleton with time or the net change in cell or nucleus volume and/or shape with time [3], 4, the current approach allows for *in situ* mapping of both the stresses to which the cells are exposed as well as the strain of the cell in response to stress, akin to an *in situ* mechanical test of a whole stem cell. On the one hand, although elastic modulus of stem cell has been previously reported using magnetic twisting cytometry [54], our method allows for the unprecedented *in situ* measurement-based parametric estimation of the model embryonic mesenchymal stem cell's elastic modulus, which shows significant anisotropy with significantly higher stiffness in compression than tension; this corroborates our previous observation of directional differences in shrinking of the same cells upon fixation [3]. On the other hand, when used in combination with methods to assess cell fate, *e.g.* gene transcription profiles [1], [3], [4], [31], [44] or fluorescent reporter constructs indicating gene activity *in situ* [55], our *in situ* live cell strain measurement method allows us to probe the stem cell's “mechanome,” enabling the probing of structure - function relationships and unfolding of lineage commitment in live model embryonic mesenchymal stem cells.

A next key step in probing the stem cell's mechanome is the elucidation of mechanotransduction mechanisms underlying stress-strain-fate relationships. For example in mesenchymal stem cells, the RhoA and RhoA kinase (ROCK) signaling pathway has been shown to control cell roundness or spreading of mesenchymal stem cells; spread cells show higher RhoA and ROCK activity than round cells and is associated with osteogenic lineage commitment even in presence of adipogenic differentiation media. In contrast, abrogation of RhoA and RhoA kinase is associated with adipogenic lineage commitment even in presence of osteogenic differentiation media. [56] Furthermore, control of MSC lineage commitment along chondrogenic or smooth muscle cell (SMC) also depends on cell shape in the presence of TGFß3 [57]. Finally, recent studies implicate the nonmotile, primary cilium, a “conserved microtubule-based organelle,” present in most eukaryotic cells, “that grow[s] from basal bodies (a centrosome-derived structure) and protrude from the cell surface,” [58], [59] for mechanosensation in most eukaryotic cells including uncommitted mesenchymal stem cells [58], [60], [61]. The approach presented here may provide new avenues to assess changes in stem cell structure and function during lineage commitment *e.g.* in association with measures of cell-cell and matricellular junctional protein expression, which will determine force balances dictating strain and shape at cell boundaries [46], as well as emergence of cytoskeletal anisotropy [3], [4], including the actin, tubulin organization at the length scale of the cell and below (axoneme) [62].

While the choice of Con A to coat the microbeads offered distinct advantages for testing the mechanome of the model embryonic mesenchymal stem cells, as delineated above, a limitation of our method relates to the ranges of flow rates applicable for this particular protein. For example, for studies in which very high flow rates are applied to cells (of particular interest for terminally differentiated cells of the vascular system), it would be desirable to use a protein with stronger binding affinity. Conversely, microbead displacements were not observable at flow rates of 0.5 dyn/cm^2^ and below, a limitation unrelated to Con A binding affinity. Similarly, tracking the displacement of microspheres for the μ-PIV method is only feasible for a range of low flow rates due to the small size of the field of view (300 µm×300 µm) and the time needed to acquire a confocal image. Whereas the limitation related to Con A binding affinity can potentially be addressed using alternative protein coatings, limitations related to the experimental set up and microscope limitations are typical for any mechanical testing scenario, where appropriate strain measurement (*e.g.* LVDT) and load cell selection are paramount.

**The studies show significant correlations between live stem cell stress - strain relationships and lineage commitment. Although correlation does not equal causation, the method presented here provides a novel means to probe the live stem cell's mechanome, enabling mechanistic studies of the role of mechanics in lineage commitment** ***as it unfolds*** **.**

## Supporting Information

Figure S1 Validation of computational predictions, comparing the velocity gradient (the slope of each curve, a measure of shear rate), a critical parameter to determine local shear stresses. (**A**) Computational flow predictions for a target wall shear stress of 0.2 dyn/cm^2^. The velocity gradient in absence of cells, as well as in the presence of cells at densities including 16,500 cells/cm^2^ (low density), 35,000 cells/cm^2^ (high), and 86500 cells/cm^2^ (very high), shows a shear rate of 17.8/s, 10.6/s, 11.76/s, and 13.2/s, respectively (R^2^>0.9). (**B**) Comparison of computational predictions to μ-PIV measurements (**D**) as well as the target wall shear stress, 0.2 dyn/cm^2^. Shear rate without cell, 16,500 cells/cm^2^, 35,000 cells/cm^2^, and 86,500 cells/cm^2^, shows 20.6/s, 6.9/s, 12.27/s, and 12.2/s, respectively (R^2^>0.9). (**C**) Computational flow predictions with target wall shear stress, 1 dyn/cm^2^. Shear rate without cell, 16500 cells/cm^2^, 35000 cells/cm^2^, and 86,500 cells/cm^2^, shows 89.3/s, 53.47/s, 54.95/s, and 64.94/s, respectively (R^2^>0.9). (**D**) Results from μ-PIV based on flow regimes delivered to achieve target wall shear stress of 0.2 dyn/cm^2^. Velocity gradient in absence of cells and in presence of cells seeded at 16,500 cells/cm^2^, 35,000 cells/cm^2^, and 86,500 cells/cm^2^, shows 19.8/s, 7.8/s, 13.2/s, and 9.9/s, respectively (R^2^>0.9). Error bars indicate standard errors and n = 3.
(TIF)
